# Peer Support for Chronic Pain in Online Health Communities: Quantitative Study on the Dynamics of Social Interactions in a Chronic Pain Forum

**DOI:** 10.2196/45858

**Published:** 2024-09-05

**Authors:** Aaron Necaise, Mary Jean Amon

**Affiliations:** 1 School of Modeling, Simulation, and Training University of Central Florida Orlando, FL United States; 2 Department of Informatics Luddy School of Informatics, Computing, and Engineering Indiana University Bloomington Bloomington, IN United States

**Keywords:** social media, chronic pain, peer support, sentiment analysis, wavelet analysis, nonlinear dynamics, growth curve modeling, online health communities, affective synchrony

## Abstract

**Background:**

Peer support for chronic pain is increasingly taking place on social media via social networking communities. Several theories on the development and maintenance of chronic pain highlight how rumination, catastrophizing, and negative social interactions can contribute to poor health outcomes. However, little is known regarding the role web-based health discussions play in the development of negative versus positive health attitudes relevant to chronic pain.

**Objective:**

This study aims to investigate how participation in online peer-to-peer support communities influenced pain expressions by examining how the sentiment of user language evolved in response to peer interactions.

**Methods:**

We collected the comment histories of 199 randomly sampled Reddit (Reddit, Inc) users who were active in a popular peer-to-peer chronic pain support community over 10 years. A total of 2 separate natural language processing methods were compared to calculate the sentiment of user comments on the forum (N=73,876). We then modeled the trajectories of users’ language sentiment using mixed-effects growth curve modeling and measured the degree to which users affectively synchronized with their peers using bivariate wavelet analysis.

**Results:**

In comparison to a shuffled baseline, we found evidence that users entrained their language sentiment to match the language of community members they interacted with (*t*_198_=4.02; *P*<.001; Cohen *d*=0.40). This synchrony was most apparent in low-frequency sentiment changes unfolding over hundreds of interactions as opposed to reactionary changes occurring from comment to comment (*F*_2,198_=17.70; *P*<.001). We also observed a significant trend in sentiment across all users (β=–.02; *P*=.003), with users increasingly using more negative language as they continued to interact with the community. Notably, there was a significant interaction between affective synchrony and community tenure (β=.02; *P*=.02), such that greater affective synchrony was associated with negative sentiment trajectories among short-term users and positive sentiment trajectories among long-term users.

**Conclusions:**

Our results are consistent with the social communication model of pain, which describes how social interactions can influence the expression of pain symptoms. The difference in long-term versus short-term affective synchrony observed between community members suggests a process of emotional coregulation and social learning. Participating in health discussions on Reddit appears to be associated with both negative *and* positive changes in sentiment depending on *how* individual users interacted with their peers. Thus, in addition to characterizing the sentiment dynamics existing within online chronic pain communities, our work provides insight into the potential benefits and drawbacks of relying on support communities organized on social media platforms.

## Introduction

### Background

The National Institutes of Health describes chronic pain as pain persisting for >3 to 6 months [1,2], which is substantially longer than a “typical” acute response to injury [3]. Chronic pain can be attributed to a wide variety of underlying medical conditions [3], and it is one of the most common health complaints in the United States [4,5]. A recent report from the National Center for Health Statistics found that the prevalence of chronic pain among adults in the United States was approximately 20%, while an additional 7.4% of survey respondents reported pain severe enough to impact daily functioning [4,5]. Moreover, prevalence rates are similarly high in high-income [6-9] and low- and middle-income countries [9,10] throughout the world, contributing to a global public health problem [11,12].

Due to the complexities of pain processing, successful management often necessitates a multifaceted approach personalized to meet the needs of the individual [13]. Chronic pain is considered to be a complex process, whereby psychosocial factors such as emotions, expectations, and social relationships interact with an individual’s physical health to contribute to the maintenance of symptoms [3,14,15]. Thus, it is encouraged to engage in health self-management strategies that contribute to mental and physical well-being [16-18]. One particularly impactful self-management strategy is participation in peer support, which occurs when individuals facing similar health challenges exchange advice, emotional validation, and educational resources [19-22]. Those experiencing chronic pain frequently report emotional distress and feelings of social isolation, and peer support can help alleviate these stressors [23].

Peer support, and pain self-management [23] more broadly, is increasingly taking place on social media platforms such as Reddit (Reddit, Inc), Facebook (Meta Platforms, Inc), and Instagram (Meta Platforms, Inc) [24]. Social media users with chronic health conditions frequently self-organize into large peer-to-peer communities that function as social and informational hubs [25]. For example, there are many peer-driven support communities on Reddit in which tens of thousands of members share private health information and respond to medical inquiries. Support interactions on social media platforms are unique in that they consist of naturally occurring discussions driven by users themselves, contrasting formally organized support groups [26] and structured web-based interventions [27]. Although users tend to have positive experiences with online support [28], interactions on the internet are not universally positive. As demonstrated by the COVID-19 pandemic, social media platforms have the potential to spread misinformation and hostility toward medical experts [29,30]. Thus, although writing about pain [31] and having supportive relationships [32] can benefit mental health, it is unclear how naturally occurring web-based health interactions influence chronic pain [24,33,34].

It is reasonable to assume that the impact of online peer support not only depends on the amount of support an individual receives but also on the affective qualities of their social interactions [14,35]. Negative affective thoughts and pain catastrophizing are theorized to contribute to the maintenance of chronic pain (eg, [3,14,36-38]), and social interactions can drive users to adopt or reject those beliefs [14]. Furthermore, the manner in which people communicate about their experiences plays an important role in shaping their emotions and expectations for the future [31,39]. It follows that health communities perpetuating overly pessimistic attitudes or hostility toward health care providers can be harmful to well-being, just as those providing emotional validation can be beneficial [22]. Thus, examining the affective qualities of web-based interactions using sentiment analysis may serve as an entry point to understanding how health is influenced by participation in online health communities (OHCs). Moreover, the analysis of online health discussions can provide broader insight into how social media users experiencing chronic pain communicate about their pain.

### Related Work

#### Psychosocial Determinants of Chronic Pain

The biopsychosocial model is the prevailing approach to understanding chronic pain [14,15,40]. From this perspective, chronic pain results from the dynamic interactions among biological, psychological, and social processes [40,41]. Edwards et al [14] provided an overview of the most widely researched psychosocial factors associated with chronic pain, centering around negative affect and pain catastrophizing (ie, negative rumination regarding pain symptoms). Negative affective thoughts are associated with an increased risk of developing chronic pain [42-44] and are believed to contribute to a variety of negative health outcomes [3,14]. Several longitudinal studies have reported that psychological distress, pain catastrophizing, and self-efficacy were moderators between pain and disability [37,38,45], such that individuals with greater negative affect were more likely to report impairment at follow-up. Overall, there is substantial evidence that expectations and emotions toward pain can impact health outcomes.

Although the factors outlined earlier are primarily psychological, they are also inextricably linked to social interactions. Maintaining strong support systems provides benefits to both physical and mental well-being [46-49], such as being associated with reduced psychological distress [50-52]. Communication with physicians, friends, or family can drive specific health beliefs that are relevant to pain outcomes. For example, individuals who experience higher spousal autonomy support report increased need satisfaction and well-being [53], while interventions teaching couples how to communicate supportively reduce pain catastrophizing [54,55]. With respect to the specific mechanisms, social interactions can perpetuate realistic treatment expectations, provide supportive (vs adversarial or solicitous [14]) feedback in response to pain expressions, and encourage health self-management [24]. Thus, social interactions are potentially facilitative or harmful depending on their qualities.

Building on the biopsychosocial approach to understanding pain, several theories have emerged that present a more explicit account of the social determinants of pain, for example, the study by Craig [56], the study by Hadjistavropoulos et al [57], and the study by Sullivan [58]. The social communication model of pain (SCMP) is an example of conceptualizing the specific biopsychosocial interactions underlying chronic pain [56,59]. The SCMP distinguishes between the effects of intrapersonal (eg, genetic predispositions and family history) and interpersonal (eg, social interactions and medical treatments) processes, emphasizing the contributions of “others” on health. According to this model, a pain-relevant social interaction occurs when a person in pain expresses their symptoms verbally or nonverbally, and an observer decodes and acts on that expression. The action taken by the observer can range from helpful to exacerbating, and it directly influences how the person in pain interprets their pain in the future. Notably, this model emphasizes that an observer’s decoding of a pain expression is biased by their own background, and the subsequent actions they take depend on their relationship to the person in pain (ie, they might be a caregiver, friend, or stranger). This framing helps contextualize online support interactions where communication tends to be anonymous, expressions of pain are text based, and observers have little obligation to respond ethically compared to “real-life” associates [60-62]. The description of social influence provided by the SCMP can also be extended to the context of social media. That is to say that a social media user’s interpretation of their pain may be iteratively updated by the feedback they receive on the web leading to changes in how they perceive and express their pain in the future. Thus, examining online health discussions is an avenue for investigating the social determinants of pain described by the SCMP.

#### Chronic Pain Support Interactions on Social Media

Social media is a term used to describe a collection of digital technologies that allow users to maintain a web-based presence, communicate and network with others, and share user-generated content [63,64]. The potential utility of social media platforms for health care purposes was recognized long before the rise of modern social networking sites, with initial research often focusing on peer-to-peer support occurring in chat rooms and messaging boards throughout the internet [65-67]. A review published in 2004 by Eysenbach et al [68] reported a lack of evidence to suggest that these early-forming OHCs had positive impacts on well-being, but the authors acknowledged that they were largely unmoderated and in the infancy of their development. With expanded access to the internet and the introduction of popular social networking sites (ie, Facebook and YouTube [Google LLC]), the growth of OHCs accelerated [69]. There is now a huge selection of communities available to social media users depending on their individual needs and preferences, varying with the amount of professional input provided [70], type of communication, and degree of anonymity [26].

Qualitative research examining pain discourse on social media platforms frequently highlights interactions containing positive emotional messages and information sharing [28,71-73]. In an analysis of 44 blogs collected across several websites, commenters overwhelmingly replied to pain blogs with messages of consolation and encouragement [73]. Furthermore, there was a “virtual online support sequence” underlying these interactions, in which commenters replied to blog posts with personal anecdotes and used their common experiences as an opportunity for emotional validation [73]. Similarly, a clinical study using semistructured interviews to gather perceptions about the use of social media platforms for pain self-management found that participants appreciated the ability to connect with others, share their personal experiences, and learn directly from their peers [28]. The perceived benefits of online peer support are not specific to chronic pain, as similar themes of emotional support, connectivity, and experiential knowledge sharing have been identified in online communities organized for a variety of chronic health conditions [19,25,74-76].

Quantitative studies investigating the effects of online support on chronic pain have been comparatively sparse and, in many cases, focus on the impact of structured intervention programs (eg, [77-79]) as opposed to the interactions occurring naturally among social media users. There is some evidence that the positive psychosocial benefits provided by traditional support groups can also be provided by casual web-based interactions, particularly when it comes to information sharing [80-82]. For example, individuals who were assigned to follow a Twitter (subsequently rebranded as X; X Corp) profile posting information about self-management strategies reported small improvements in pain, emotional distress, and quality of life after 6 months of virtual interactions [81]. When considering chronic illnesses more broadly, online peer support has been found to reduce feelings of isolation in adolescents [83], and the mere act of self-disclosing about life stressors (ie, not just chronic health) can reduce emotional distress [84]. Although peer support on social media platforms can play a beneficial role, these effects likely depend on the specific social dynamics in the community, such as the presence of peer role models to help guide conversations [85] or the type of messages that are circulated. Web-based health resources will be even more popular among future generations [24], highlighting the need for continued research on web-based health self-management. Specifically, social media research can lead to an improved understanding of how individuals experiencing chronic pain develop their health-related beliefs.

### Objectives

This study investigated peer support interactions in OHCs by focusing on the sentiment of individual support interactions. Applying sentiment analysis to the comment histories of users in a popular chronic pain forum on Reddit, we measured the degree to which users synchronized the sentiment of their comments to match their peers, and we modeled the trajectories of their sentiment over the course of their community participation. This work addresses 3 questions regarding the use of online peer support for chronic pain: Do users who engage in online peer support synchronize their pain expressions to match the language of other community members? How does a user’s sentiment progress over time in response to online support interactions? And finally, how do specific interaction dynamics, such as affective synchrony with other users, relate to changes in sentiment?

## Methods

### Study Design

We analyzed comments posted to a popular chronic pain support community on Reddit. Reddit is a platform where users can create personalized forums dedicated to discussing specific topics [86] and within each user-created forum (ie, “subreddit”), users define their own rules regarding content and membership. The subreddit analyzed in this study describes itself as a forum for users to discuss their conditions with their peers and share advice; however, we have opted to withhold the exact name of the community out of concern for user privacy. The community guidelines provided by the moderators discourage direct medical advice and suggest consultation with professionals before participation. However, it is unclear how strictly these guidelines are enforced. In terms of content, discussion threads contain a mixture of advice-seeking, informational resources, and personal anecdotes. A recent paper using latent Dirichlet allocation to analyze chronic pain subreddits on Reddit similar to the one used in this study found that users most frequently mentioned phrases related to lower back pain in their posts in addition to words such as “doctors,” “help,” and “work” [87], providing evidence that discussions within these communities are highly focused on the topic of chronic pain.

We collected the post histories of 200 randomly selected Reddit users active on the subreddit and calculated the sentiment of their comments using a dictionary-based approach [88]. Sentiment analysis is a common natural language processing method used to classify and describe the emotional expressiveness of text by analyzing the valence, intensity, and structural features of language [89,90]. Next, we used bivariate wavelet analysis to estimate the degree of sentiment synchrony between users during interactions, which is a technique popularized for its applications on complex systems [91]. Unlike a standard linear approach (eg, cross-correlation), wavelet analysis is robust against nonstationarity and describes multiple types of synchronies, including matched intensity, comovement, and leader-follower dynamics [91,92]. Moreover, wavelet analysis can effectively separate changes in sentiment that are occurring reactively (eg, comment to comment), from those occurring globally over hundreds of interactions. Finally, we examined whether discourse on the subreddit was associated with negative or positive changes in the health attitudes of users by using growth curve analysis to model trajectories in comment sentiment.

### Ethical Considerations

This study was reviewed by the institutional review board at the University of Central Florida (00001430). Observational public data from Reddit were collected, and there were no direct interactions with human participants. Reddit is a social media platform where users typically provide pseudonyms in place of their real names, which are not reported, and we did not collect personally identifiable information. We randomly sampled users who fit our eligibility criteria, meaning a particular profile’s inclusion is not discernable. In addition, we have opted to withhold the name of the subreddit that we collected data from to further obfuscate the identity of its members. Given the large number of comments we collected, it was possible that some of the text contained identifiable information indirectly in the form of self-disclosures. To protect user anonymity, each user in our sample received a randomized identifier in place of their Reddit username, and the text content of their comments was aggregated using the procedures detailed in the sections that follow such that it would not be possible to link the stored data to any individual Reddit user or real-life individual.

### Data Sampling

We collected publicly available Reddit data using the Pushshift database [93] and Reddit’s application programming interface [94]. On Reddit, the term *submission* refers to user-created discussion threads posted within each community, while *comments* refer to the text replies within each discussion thread. To be included in our final sample, users had to be moderately active members of the support subreddit, with at least 100 total comments and submissions after excluding self-deleted posts and those deleted through moderation. This minimum activity threshold was applied to ensure there was an adequate number of data points for wavelet analysis at lower frequencies [92]. In addition, we excluded users serving as moderators and Reddit profiles posting automatic replies (eg, apparent bot accounts). To identify Reddit profiles meeting this criterion, we downloaded every publicly available comment and submission made to the subreddit in the last 10 years. We then calculated each user’s total number of posts in the community and randomly selected 200 users with more than the minimum required activity. The sample size was determined using an a priori power analysis for a growth curve model with a small effect size, *f*^2^=0.10, and α=.05, estimated based on similar previous research examining sentiment in online discussion forums [95-97]. Finally, we collected data from the public interactions these users had on Reddit. We define an interaction as the pairing between a user’s personal comment and the post they were replying to when making that comment. For example, an interaction could involve a user posting a top-level comment in response to a discussion thread. An interaction could also involve posting a reply to someone else’s comment inside of a discussion thread. Thus, for each user, we collected the following: (1) the text of all their comments, (2) the corresponding text of posts they interacted with, (3) comment scores (upvotes minus downvotes), and (4) time of posting.

### Data Processing

Each user’s comment history was formatted as a time series sorted in chronological order, and each point in those time series represented 1 interaction with the community. Activity occurring outside of the subreddit of interest was excluded from the analysis. In addition, the Reddit application programming interface returns an error message instead of the original text when comments are removed by moderators or users themselves, and these error messages were excluded. A small portion of comments contained single-word phrases, such as a web address or emoji. Sentiment calculations for these items would have been unreliable, and we opted to remove comments containing <3 words. A total of 73,876 of comment interactions were collected from the pain support subreddit. Of those comments, 5670 (7.68%) were excluded based on low word count or missing data. Shortly before analysis, we also discovered 1 user acting as a moderator, and this individual’s data were removed due to their unique role in the community. Thus, our final sample size included 68,206 (92.32%) of the 73,876 peer-to-peer interactions from 199 users and across 29,360 unique discussion threads.

After cleaning the data, we calculated sentiment scores using a lexicon-based approach [88], such that there were separate scores for both sides of each social interaction. To improve the reliability of these estimates, we compared 2 popular techniques in the psychology and social media literature. The Linguistic Inquiry and Word Count (LIWC) [98] dictionary is a natural language processing tool used to extract psychological language attributes and has been applied in a variety of digital health and social media contexts (eg, [99-102]). The LIWC dictionary calculates the percentage of text pertaining to specific topics (eg, the percentage of health-related words) and provides summary variables measuring higher-level cognitive processes. For our purposes, we used the LIWC dictionary to calculate sentiment, number of words, and percentage of health-related words in each comment. Sentiment scores from the LIWC dictionary ranged from –100 to 100, with higher scores indicative of more positive language, lower scores indicative of more negative language, and scores around 0 indicative of neutral language [98,103]. For comparison, we also calculated sentiment using the Valence Aware Dictionary for Sentiment Reasoning (VADER) [104]. Sentiment scores from VADER have been validated against manual human reviewers and were designed for the analysis of brief web-based internet interactions [104]. Like the LIWC dictionary, compound sentiment scores from VADER ranged from –1 to +1, with higher scores indicative of more positive language, lower scores indicative of more negative language, and scores around 0 indicative of neutral language [105]. We found both methods were highly consistent (α=.93) and strongly correlated (*r*=0.70; *P*<.001); consequently, we created a compound score by standardizing and averaging the results of both. Generating compound scores through aggregation has been shown to improve reliability [106], and, in our case, this approach minimized the influence of text passages where the 2 methods diverged. As a result, each user had one-time series representing their personal comment sentiment and one-time series for the sentiment of comments they interacted with when making their personal comments.

### Bivariate Wavelet Analysis

Bivariate wavelet analysis was used to quantify the sentiment synchrony between users and their peers. We used the cross-wavelet transform (XWT) and wavelet coherence (WTC) to gain information about matching sentiment intensity and phase-locked (ie, correlated) changes in sentiment. Compared to cross-correlation, wavelet analysis describes changes in sentiment at multiple frequencies (eg, short- vs long-term changes in sentiment). This allowed us to isolate fluctuations in sentiment occurring comment to comment (ie, high-frequency changes) from those occurring over dozens or hundreds of interactions (ie, lower-frequency changes). In other words, this analysis provided insight into short-term reactionary changes in sentiment versus long-term global changes. We briefly discuss XWT and WTC subsequently; however, readers should refer to the work of Torrence and Compo [107] for a comprehensive guide to wavelet analysis or Issartel et al [92] for applications on behavioral synchrony. A discussion of the method and theoretical implications in the context of social media is provided by Necaise et al [95].

**Figure 1:**
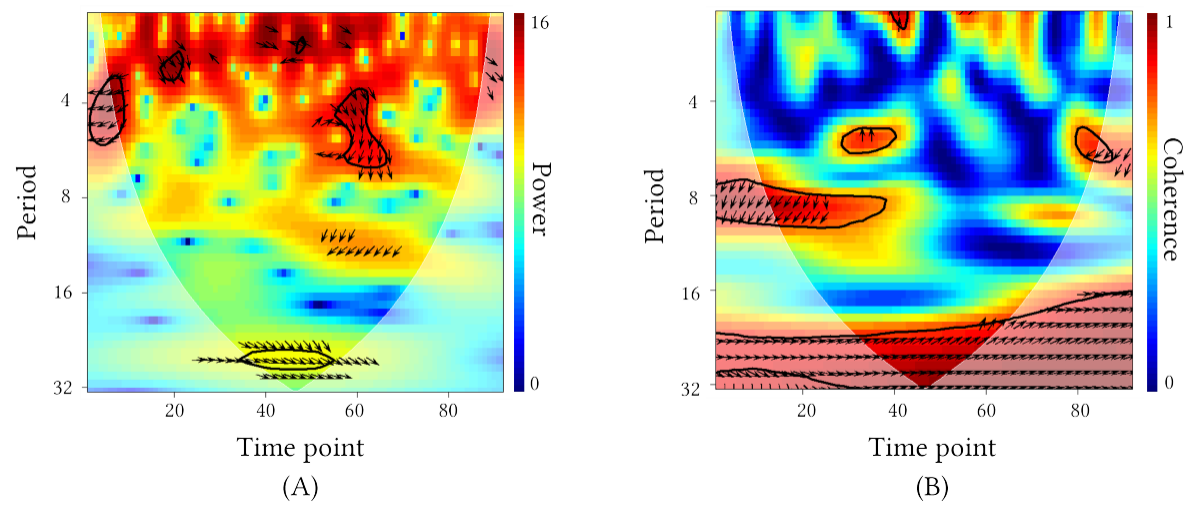
Example cross-wavelet spectrum plots visualizing (A) high common power (cross-wavelet transform [XWT]) and (B) coherence (wavelet coherence [WTC]) generated from 1 user’s sentiment compared to the sentiment of posts they replied to. The XWT identified significant regions across 2 signals with similarly intense sentiment fluctuations. The WTC plot identified regions across the 2 signals having high coherence. Finally, the relative phase (RP) angle is visualized as arrows within significant regions of the cross-wavelet plots. Arrows pointing right represent in-phase fluctuations, while arrows to the left represent antiphase fluctuations. The common power and coherence were calculated as the percentage of their respective plots having significant high common power or coherence (regions circled in black).

Wavelet calculations were completed using the *biwavelet* package in R (R Foundation for Statistical Computing) [108] with a Morlet wavelet function (*ω*_0_=6). The Morlet wavelet was selected because it provides good resolution compared to other functions [107]. Furthermore, cross-wavelet calculations can be sensitive to large spikes in the data, so the data were transformed using a cumulative distribution function, so the values represented percentiles. This transformation has been suggested in previous literature [91]. The XWT was applied using the method outlined by Grinsted et al [91] and identified points in time (x-axis of Figure 1) and frequency (“period” on the y-axis of Figure 1) where users had fluctuations in sentiment that were similar in intensity as their conversation partners. Significance is determined in comparison to AR(1) background processes [91,92], and the significant points (circled in black in panel A of Figure 1) are referred to as regions of *high common power* [91]. The XWT also provided information about the *relative phase (RP) relationship* between users and their conversation partners within regions of high common power (depicted by the orientation of arrows in Figure 1). The RP angle was extracted from the XWT and described how user sentiment changed relative to their peers [91], providing information about leader-follower dynamics.

In addition to measuring common power with the XWT, we also calculated user *coherence* with their peers using WTC. The formula for WTC is similar to Pearson correlation except localized in frequency and time [91]. Therefore, it is helpful to think about coherence as an indicator of correlation or “comovement” between 2 signals. We used a standard smoothing factor of 0.6 for Morlet wavelets in the WTC calculation and tested for significant coherence against simulated data via Monte Carlo methods with 2000 random initializations [91]. Regions of significant coherence are circled in black in panel B of Figure 1.

### Statistical Approach

#### Measuring Sentiment Synchrony

Several metrics can be extracted from cross-wavelet power plots relevant to describing synchrony [92] (Table 1). We were interested in global estimates of synchrony and therefore calculated the percentage of each user’s cross-wavelet plot exhibiting significant common power and coherence (Figure 1). Likewise, we calculated the mean circular angle [109] of the RP relationships in regions with significantly high common power. These calculations excluded points outside of the cone of influence where estimates can be unreliable, as depicted by the lightened regions in Figure 1. Each of these metrics was used to describe synchrony from a distinct perspective. Social media users with higher common power were more synchronous with their peers in terms of matched intensity, and higher coherence was an indicator of correlation in time-frequency space. RP angle describes *how* the sentiment of a user changed directionally with respect to the community. An RP angle of 0° would indicate the sentiments of a user and their peers were *in-phase* (ie, by fluctuating up and down at the same time), while an RP angle of 180° would indicate an *antiphase* relationship (ie, by alternating opposite of one another).

**Table 1:** Description of synchrony metrics derived from wavelet analysis, the label provided to them in the text, and a description of which aspect of synchrony each outcome measures.

Synchrony measures	Calculation	What it measures
Common power	Percentage of significant points in XWT^a^ plot	Degree of matched sentiment intensity in time-frequency domain
Coherence	Percentage of significant points in WTC^b^ plot	Degree of correlated behavior in time-frequency domain (eg, phase-locked behavior or comovement)
RP^c^ angle	Mean circular angle of RP in regions of significant common power	Leader-follower dynamics (eg, fluctuating in perfect synchrony versus in an alternating pattern)

^a^XWT: cross-wavelet transform.

^b^WTC: wavelet coherence.

^c^RP: relative phase.

#### Testing for Significant Synchrony Against Shuffled Baseline

To determine whether our estimates of common power and coherence constituted a significant amount of synchrony beyond what could be explained by random variance, we repeated the wavelet estimations described above on a shuffled version of each user’s data. We then used paired 2-tailed *t* tests to compare common power and coherence from the original data to the shuffled baseline. It should be noted that our calculations for common power and coherence were based on the percentage of significant points across all frequencies in the power spectrum, leading to deflated percentages that may be difficult to interpret. Thus, we provide effect size estimates to better depict the magnitude of differences between shuffled and real data [110].

To provide evidence of the construct validity of our wavelet-based outcome measures, we also compared common power and coherence to a more traditional synchrony estimate. Pearson correlation coefficients have been used in previous literature as global estimates of synchrony (eg, [111]), so we calculated a Pearson coefficient for each user by correlating the sentiment of their personal comments to the sentiment of their peers. We then conducted a correlation analysis between Pearson coefficients and the bivariate wavelet metrics in both the real and shuffled data.

#### Comparing Degree of Synchrony Across Different Frequencies of Change

As described previously, although there is some overlap between cross-correlation and bivariate wavelet analysis in how they describe synchrony, wavelet analysis has the added benefit of decomposing signals into individual frequency components. This allowed us to analyze synchrony at specific frequency bands (ie, slow vs fasting moving sentiment changes). In addition to extracting common power and coherence across each user’s entire cross-wavelet spectrum plot, we also calculated common power and coherence within 3 distinct frequency bands: low (fluctuations in sentiment unfolding between 20 and 32 interactions), medium (fluctuations unfolding between 10-20 interactions), and high (fluctuations unfolding in <10 interactions). We then used repeated measures ANOVA to compare the amount of synchrony present at each of these 3 frequency bands to investigate whether the degree of sentiment synchrony differed depending on the timescale.

#### Mixed-Effects Growth Curve for Modeling Sentiment Trajectories

Finally, we examined how users’ comment sentiment changed as a function of activity on the subreddit. To examine the trajectories of comment sentiment, we used the *LME4* package in R to fit a mixed-effects growth curve model. A moving window transform was applied to improve the interpretability of model coefficients. We divided each user’s data into a series of equally proportioned windows and calculated average comment sentiment within each *interaction window* such that each user had 100 data points representing 1% of their total activity. In other words, the value at interaction window 1 reflected the average sentiment over the first 1% of a user’s comments, while the value at interaction window 100 reflected the average sentiment over the last 1% of a user’s comments. This approach has been used in similar literature [112].

For the growth curve analysis, we fit a mixed-effects model with comment sentiment entered as the dependent variable, interaction window as a fixed-effect, and user ID as a random intercept. The common power estimate, average word count, average number of days active on the subreddit, and percentage of health-related words were entered as covariates. We included word count and the percentage of health-related words to control for the potentially confounding effects of abnormally long comments or medical terms on sentiment scores (ie, certain medical terms could be misclassified as negative). Furthermore, common power was included as an estimate of overall sentiment synchrony, and this allowed us to examine individual changes in sentiment independent of sentiment change related to entrainment with peers [91]. Finally, we included the number of days active in the subreddit to compare users with long versus short tenures in the community.

## Results

### Descriptive Statistics

**Figure 2:**
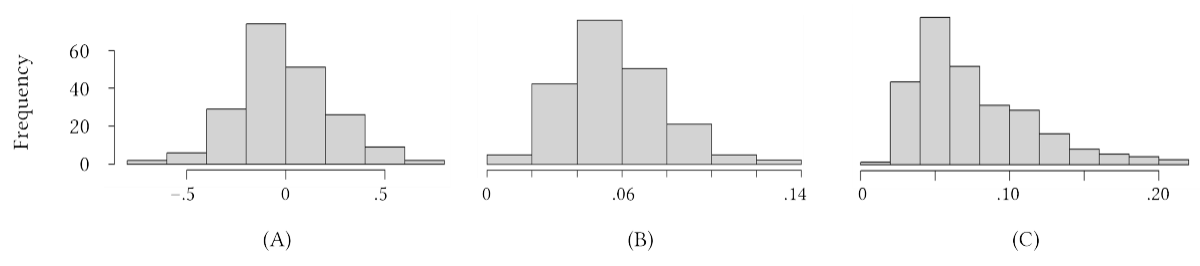
Frequency distributions for primary outcome variables, including (A) user comment sentiment, (B) common power, and (C) coherence.

Table 2 contains descriptive statistics related to the Reddit activity of users in our sample, the sentiment of their comments, feedback received from their peers, and wavelet synchrony metrics. Frequency distributions for comment sentiment, common power, and coherence can be found in Figure 2. Although we only analyzed interactions within the support community, it is worth noting users made a substantial number of posts to other subreddits. On average, activity inside of the support group constituted 22.03% (SD 24.01%) of users’ total Reddit activity.

**Table 2:** Descriptive statistics.

Variable	Value, mean (SD)
Comments made per user	371.24 (518.18)
Comments made per day	2.87 (2.09)
Number of days active in the community	141.22 (142.56)
Sentiment of personal comments	–0.01 (0.24)
Sentiment of peer comments	–0.28 (0.15)
Number of replies received from peers	200.57 (272.63)
Comment score (upvotes–downvotes)	3.17 (1.27)
Common power (%XWT^a^)	0.06 (0.02)
Coherence (%WTC^b^)	0.08 (0.04)

^a^XWT: cross-wavelet transform.

^b^WTC: wavelet coherence.

### Users Exhibited Significant Synchrony Compared to Shuffled Baseline

We found users had significantly more common power during their real support interactions (mean 0.06, SD 0.02) compared to a shuffled baseline (mean 0.04, SD 0.01; *t*_198_=4.02; *P*<.001; Cohen *d*=0.40). Likewise, there was significantly more sentiment coherence during real interactions (mean 0.08, SD 0.04) as compared to shuffled data (mean 0.05, SD 0.03; *t*_198_=6.57; *P*<.001; Cohen *d*=0.64). These results suggest users synchronized in terms of matching each other’s intensity (common power), and changes in user sentiment corresponded to changes in peer sentiment (coherence).

Comparing these results to a traditional Pearson correlation approach (Table 3), we found significant positive correlations between Pearson correlation coefficients, real-data coherence (*r*_197_=0.46; *P*<.001), and real-data common power (*r*_197_=0.18; *P*<.001). However, there were no relationships between Pearson coefficients and wavelet calculations in the shuffled data (*P*=.77 and .50). Taken together, we found evidence that users synchronized their sentiment during support interactions beyond what could be explained by random variance in their data. The results of our correlation analysis also supported the validity of our synchrony analysis.

**Table 3:** Correlation analysis relating traditional synchrony measure (Pearson correlation) to bivariate wavelet estimates (original and shuffled data)^a^.

Variable		Pearson coefficients	Coherence	Coherence shuffled	Common power	Common power shuffled
**Pearson coefficients**						
	*r*	1	0.46	–0.02	0.18	0.05
	*P* value	—^b^	<.001	.77	.01	.50
**Coherence**						
	*r*	0.46	1	0.07	0.41	–0.05
	*P* value	<.001	—	.35	<.001	.48
**Coherence shuffled**						
	*r*	–0.02	0.07	1	–0.04	0.14
	*P* value	.77	.35	—	.60	.05
**Common power**						
	*r*	0.18	0.41	–0.04	1	–0.01
	*P* value	.01	<.001	.60	—	.87
**Common power shuffled**						
	*r*	0.05	–0.05	0.14	–0.01	1
	*P* value	.50	.48	.05	.87	—

^a^Pearson correlation, listwise deletion.

^b^Not applicable.

### User Sentiment Fluctuated In-Phase With Peer Sentiment

**Figure 3:**
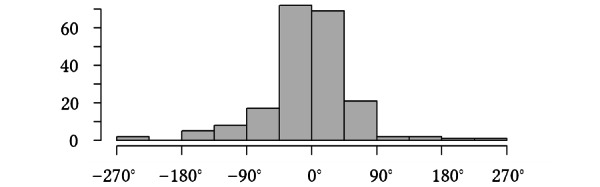
Frequency distribution for average relative phase (RP) angle by user.

Across all users, we found an average circular RP angle of 2.81°, suggesting users were *in-phase* with the sentiment of their peers during interactions (Figure 3). Thus, the sentiment intensity of users fluctuated in *the same direction* without a clear leader or follower*,* and periods of positive posting by a user were mirrored by periods of positive posting by their peers.

### Users Had Greater Synchrony in Low- Versus High-Frequency Sentiment Changes

We found that the average common power varied significantly depending on frequency (*F*_2,198_=17.70; *P*<.001). The common power in low-frequency bands (mean 0.08, SD 0.10) was significantly higher than medium- (mean 0.05, SD 0.05; *t*_199_=3.66; *P*<.001) or high-frequency bands (mean 0.05, SD 0.01; *t*_199_=5.05; *P*<.001). The same was true for coherence (*F*_2,198_=3.79; *P*=.01), as there was significantly more coherence in low (mean 0.10, SD 0.15) compared to high frequencies (mean 0.07, SD 0.03; *t*_199_=2.36; *P*=.02). There were no differences in coherence between medium and high frequencies (*P*=.13). These findings suggest that synchrony was most evident in long-term sentiment fluctuations occurring over hundreds of interactions as opposed to the more reactionary changes in sentiment occurring from comment to comment. There were no differences in RP angle (*P*=.11), indicating the sentiment of users and their peers were in-phase regardless of scale.

### Sentiment Trajectories Depended on Activity and Degree of Synchrony

**Figure 4:**
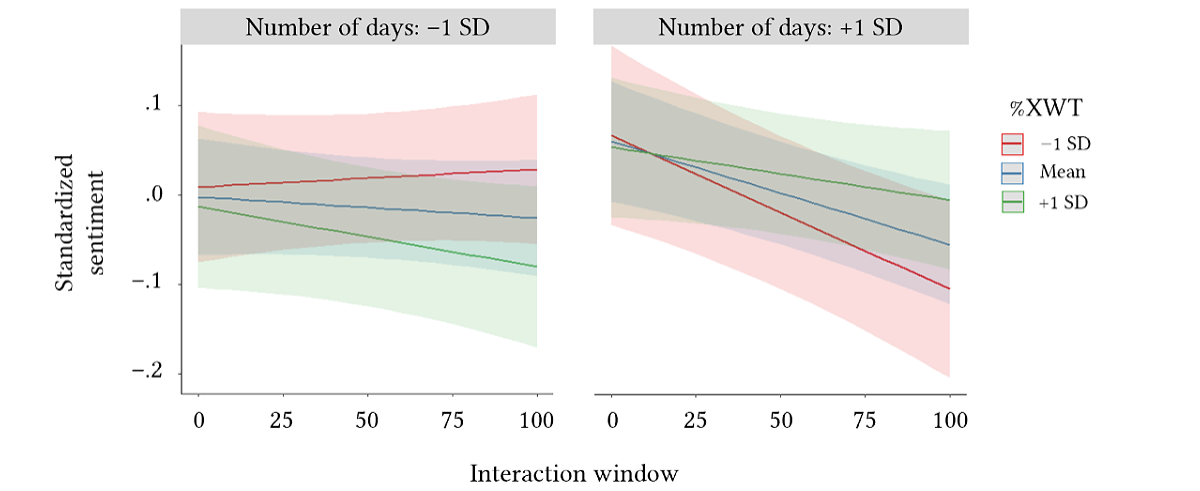
A 3-way interaction effect of common power, number of days active on subreddit, and sentiment scores. XWT: cross-wavelet transform.

Across all users, sentiment decreased significantly when interacting with the subreddit (β=–.02; *P*=.003), and this negative trend was observed while controlling for differences in average word count (β=–.10; *P*<.001) and average health-related language (β=–.20; *P*<.001; Table 4). In addition, there was a significant 3-way interaction between interaction number, synchrony, and the number of days active (β=.02; *P*=.02), indicating the trajectory of comment sentiment depended on the degree of emotional synchrony and the duration of subreddit participation. Users who were active on the subreddit for a greater number of days exhibited a steeper decline in sentiment compared to those who were active for shorter durations. Thus, sentiment not only decreased as users interacted with their peers but also decreased longitudinally with the total number of days spent seeking online support. However, this compounding effect depended on affective synchrony. Greater affective synchrony was associated with negative changes in sentiment for those who spent less time on the subreddit, but it functioned as a protective factor for those who were active for longer (Figure 4).

**Table 4:** Mixed-effects growth curve model examining the trajectory of comment sentiment and interactions with synchrony and days active^a^.

Predictors	Sentiment		
	β	Standard CI	*P* value
Intercept	–.00	–0.04 to 0.03	.80
Interaction window	–.02	–0.03 to –0.01	.003
Common power	–.01	–0.04 to 0.03	.71
Number of days active	0	–0.04 to 0.05	.74
Percent of health-related words	–.20	–0.24 to –0.16	<.001
Average word count	–.10	–0.14 to –0.06	<.001
Interaction window: common power	0	–0.01 to 0.02	.15
Interaction window: number of days	–.02	–0.03 to –0.00	.04
Common power: number of Days	.03	–0.01 to 0.07	.92
Interaction window: common power: number of days	.02	0.00 to –0.03	.02

^a^σ^2^=0.88, τ_00_ (random intercept variance)=0.07; intraclass correlation coefficient=0.07; N_author_=199; observations=19,892; marginal *R*^2^=0.05; conditional *R*^2^=0.12.

## Discussion

### Principal Findings

#### Sentiment Synchrony

We found evidence that social media users synchronized the emotion of their language during conversations within a pain support community on Reddit. Users not only matched the sentiment intensity of their peers but also exhibited significant phased-locked behavior, showing positively correlated (ie, in-phase) changes in sentiment at several different frequency scales. Synchrony during dyadic interactions is a well-documented phenomenon, as conversation partners tend to mimic each other’s emotions [113], language style [114,115], and nonverbal patterns of behaviors [111,116]. Wood et al [113] suggest that affective synchrony assists with information processing, emotional regulation, and social bonding. Under this framework, synchrony by members of the support community may reflect a more general process underlying supportive interactions, whereby participants bond over shared experiences using similarly intense emotional expressions. Synchrony is also associated with positive benefits for conversation partners, including increased feelings of social connectedness [117] and positive changes in affect after interaction [117,118]. For example, a study on online support seeking reported that greater linguistic synchrony during text-based discussions predicted satisfaction with support and improvements in mood [114]. We suspect affective synchrony in the chronic pain subreddit was an indicator of supportive (vs adversarial [14]) interactions and empathetic language [113]. Peer support aims to connect individuals who have similar medical conditions with the hope that their common experiences promote feelings of acceptance and understanding [119]. By that basic conceptualization, affective and experiential synchrony can be seen as essential elements of peer support. Although we do not collect data about health status, future research should investigate whether sentiment synchrony is predictive of positive versus negative health outcomes.

Our investigation of synchrony by timescale (ie, low, medium, and high frequencies), using the XWT, revealed that users were most synchronous with their long-term sentiment dynamics. Each data point included a single interaction between a user and one of their peers, such that low-frequency synchrony (unfolding over hundreds of interactions) represented large-scale coordination across hundreds of community members. In other words, our findings reflect a tendency of users to affectively synchronize at the level of the community over long periods as opposed to at the level of individual interactions. This tendency can be interpreted as a type of social learning, showing how repeated community interactions influence emotional expressions over long periods. Social learning occurs when people observe and imitate the behaviors of others [120], and, in the context of affective synchrony, it involves multiple people coregulating their emotions to align with a larger group [113]. It is reasonable that online community members would steadily adjust their emotional expressions as they become more familiar with group dynamics.

A concern among medical professionals in recommending web-based resources is that social media users will be negatively influenced by misinformation or the attitudes of peers [121,122]. In addition, a study on user perceptions found that users often experienced negative web-based interactions that damaged their opinions of online support [123]. By contrast, our results are a promising indicator that users were partaking in supportive and emotionally synchronous interactions. However, synchrony has the potential to be harmful if it proliferates negative health attitudes. Related to this concern, we observed in our descriptive statistics that the sentiment of users’ personal comments was more positive on average than the content they interacted with, suggesting users often interacted with content containing more negative language than their own. Affective synchrony among users highlights the importance of moderation in peer support communities for promoting positive as opposed to “toxic” (eg, [124,125]) interactions. For example, it is common for many of the pain support communities on Reddit to prohibit users from providing explicit medical advice and to encourage users to talk to their providers about their participation in the community. However, it is unclear how strictly these rules were enforced in the subreddit we analyzed or in OHCs more broadly.

#### Trajectories in User Sentiment

The biopsychosocial model of pain emphasizes how supportive relationships can promote mental well-being [14], but it is unclear how naturally occurring discussions in OHCs influence attitudes toward health. Our mixed effect growth curve model revealed a significant increase in negative sentiment over time across all users, controlling for differences in health-related language (eg, “pain” and “health”) and word count. To our knowledge, this is the first study to examine sentiment trajectories in online *chronic pain* communities. Previous research has focused primarily on mental health support, and findings from those studies have been highly variable [96,97,126]. For example, Davcheva et al [96] reported that changes in sentiment on mental health forums depended on underlying conditions, such that seeking support for anxiety and depression was associated with positive changes in sentiment, and obsessive-compulsive disorder was associated with negative changes in sentiment. This study contributes to this literature by demonstrating how aspects of social media use can further enhance online support outcomes. Specifically, affective asynchrony and community tenure predicted negative versus positive sentiment trajectories. Moreover, our findings align with the SCMP, which describes pain expressions as evolving over time in response to pain-relevant social interactions.

Given the purported emotional benefits of peer support [14,32], findings of a negative trend in sentiment across users were somewhat unexpected. Chronic pain is referred to as a “vicious cycle” [127], with physical and psychological symptoms progressing many years beyond their onset [8,41]. Several studies have indicated long-term declines in affect co-occurring with chronic illness [128-130], and psychological distress and pain are theorized to be reciprocal [14,38,40,131]. Thus, our results may have reflected the continued frustrations experienced by users during their pain self-management journey as opposed to representing any specific adverse impact of web-based interactions. The fear-avoidance (FA) model describes chronic pain as a negative affective feedback loop between catastrophizing and pain, and this “downward spiral” [132] of affect propels symptoms forward until the loop can be interrupted [133,134]. The negative sentiment trajectories observed in this study are consistent with the downward spiral of affect described by the FA model, and, aligning with this idea, users who spent the most time on the subreddit exhibited the most pronounced increases in negative sentiment. It is also important to highlight that a portion of users progressed positively in our sample despite the overall negative trend, and these individual differences were related to having lower community synchrony and shorter community tenures. By identifying the factors that contribute to positive sentiment trajectories, it may be possible to develop personalized interventions that target users based on the dynamics of their interactions. According to the FA model of chronic pain, users may benefit from information about pain catastrophizing, mental health, and social support [134]. This could be provided in the form of a “stickied” thread that remains at the top of the forum or as an automated message sent to members.

Finally, affective synchrony emerged as a significant factor in predicting positive versus negative sentiment outcomes. For long-tenured community members, synchrony appeared to function as a protective factor against increased negativity. This is consistent with research conceptualizing synchrony as a process promoting social connectedness and emotional support [113,114]. In other words, we would expect individuals involved in more supportive interactions to be more emotionally resilient [135,136]. It is less clear as to why synchrony was dually associated with negative sentiment trajectories among *short*-tenured community members. One possibility is that these members differed in the type of support they were seeking. There was a significant correlation between the number of days active on the subreddit and word count, *r*_197_=0.18; *P*<.001, indicating that short-tenured members were involved in less verbose discussions. It is possible these users were seeking a type of support in which affective synchrony was irrelevant, such as by seeking occasional medical information as opposed to the emotional validation achieved by sharing personal narratives (eg, [76]).

### Strengths and Limitations

Our findings make several contributions to the literature. First, our study is unique in quantitatively examining the dynamics of online chronic pain support communities, contrasting the current literature that is primarily qualitative and focused on pain narratives [28,73,80,87,123,137,138]. Second, the qualities of pain are typically investigated through measures of central tendency (ie, by comparing average pain catastrophizing across groups). However, the biopsychosocial model describes chronic pain as a dynamic (changing over time) and complex (evolving from the interactions of multiple factors) process [14,139,140]. Consistent with this framing of chronic pain, we used wavelet analysis to analyze sentiment synchrony, which is an analytic technique better suited to describe complex system interactions and nonstationary behavior [92,107]. Next, previous studies of online pain support groups have often relied on small sample sizes and do not analyze data longitudinally. In comparison, we analyzed the entire comment histories of Reddit users consisting of over a million comments and approximately 70,000 interactions within 1 OHC. Thus, we present a comprehensive view of users’ web-based interactions inside the chronic pain subreddit over nearly a decade. Finally, as opposed to examining the impact of a structured intervention (eg, [27,85]), we examined naturally occurring conversations that are more representative of everyday social interactions. Unlike a structured intervention or professionally managed support group, our analyses considered undirected web-based conversations where participants likely encountered a wide variety of positive and negative actors. Participants were free to express their emotions with little inhibition related to research participation, especially due to the relatively anonymous nature of Reddit [33]. This type of longitudinal and naturalistic look into an individual’s pain expressions would be exceedingly difficult to collect in traditional settings [33].

Despite the strengths of our quantitative approach, there are several limitations. Notably, our investigations lacked data about user health outcomes. We investigated text sentiment as a correlate to chronic pain attitudes and emotions, and our results provided novel insights regarding how sentiment progressed in 1 OHC. However, without data on health outcomes, we cannot know if these trends were indicative of declines in physical or psychological well-being. Users could have viewed the forum as an opportunity for emotional catharsis, venting about their pain increasingly over time. There are some circumstances where negative emotional expression can lead to positive outcomes [141-143], particularly if those expressions are positively reframed by the audience [144]. In contrast, emotional catharsis can be counterproductive and lead to agitation [145,146]. Thus, it is necessary to exercise caution when drawing conclusions about the merits of support seeking on social media platforms based on the findings of this study.

Another potential limitation relates to our selection of Reddit as a data source. Reddit blends features from popular social networking sites (eg, the ability to personalize a user profile, add friends, or form groups) with the formatting of a web-based discussion board. As a result, the interactions on Reddit tend to surround topical discussions as opposed to resharing or reposting user-generated content. This format facilitates verbose and focused conversations, and it allows us to collect rich longitudinal data about pain-related social interactions. Reddit also affords users increased anonymity compared to social networking sites such as Facebook, as a user’s Reddit profile is not tied to their identity, friends, or family. There is evidence to suggest that social media users seeking chronic pain support prefer the ability to remain anonymous [28], thus, we suspected users would more candidly share their feelings on Reddit compared to a public-facing social networking site. However, given the unique features of Reddit, it is not clear how our findings generalize to more popular social media platforms that promote different styles of communication. It is also not clear if our findings concerning chronic pain support can be applied to OHCs for other types of chronic health conditions.

### Conclusions

Although social media provides easy access to massive peer support networks, it also has the potential to spread negative attitudes and beliefs about pain. We found evidence that social media users synchronized the emotional intensity of their language during virtual conversations about chronic pain, which can be viewed as an indicator of social bonding [113] and highlights the influence of OHC interactions on pain expressions. Furthermore, affective synchrony functioned as a protective factor against continued negative language use among those with the most negative expressions of pain in the online community. Despite these promising findings, we noted a steady increase in negative sentiment averaged across users as they continued their community participation. This negative trend is consistent with the “downward spiral” of affect described by the FA model of pain [132], suggesting that the impact of OHCs on pain expressions varies to a large degree dependent on specific user behaviors. Our results highlight the importance of considering specific user and community dynamics when assessing the impact of OHCs. Furthermore, our synchrony indices show there is a high degree of peer influence on pain attitudes and expressions, consistent with the SCMP.

## Abbreviations

FA: fear-avoidance
LIWC: Linguistic Inquiry and Word Count
OHC: online health community
RP: relative phase
SCMP: social communication model of pain
VADER: Valence Aware Dictionary for Sentiment Reasoning
WTC: wavelet coherence
XWT: cross-wavelet transform

## Data Availability

The data generated during and analyzed during this study are not publicly available due to the developer data terms provided by Reddit, which prevents us from distributing or resharing data collected using the Reddit application programming interface.

## References

[1] Chronic pain: what you need to know. National Institutes of Health National Center for Complementary and Integrative Health.

[2] Pain. National Institutes of Health National Institute of Neurological Disorders and Stroke.

[3] Cohen SP, Vase L, Hooten WM (2021). Chronic pain: an update on burden, best practices, and new advances. Lancet.

[4] Zelaya CE, Dahlhamer JM, Lucas JW, Connor EM (2020). Chronic pain and high-impact chronic pain among U.S. adults, 2019. NCHS Data Brief.

[5] Yong RJ, Mullins PM, Bhattacharyya N (2022). Prevalence of chronic pain among adults in the United States. Pain.

[6] Almalki MT, BinBaz SS, Alamri SS, Alghamdi HH, El-Kabbani AO, Al Mulhem AA, Alzubaidi SA, Altowairqi AT, Alrbeeai HA, Alharthi WM, Alswat KA (2019). Prevalence of chronic pain and high-impact chronic pain in Saudi Arabia. Saudi Med J.

[7] Rustøen T, Wahl AK, Hanestad BR, Lerdal A, Paul S, Miaskowski C (2004). Prevalence and characteristics of chronic pain in the general Norwegian population. Eur J Pain.

[8] Tunks ER, Crook J, Weir R (2008). Epidemiology of chronic pain with psychological comorbidity: prevalence, risk, course, and prognosis. Can J Psychiatry.

[9] Sá KN, Moreira L, Baptista AF, Yeng LT, Teixeira MJ, Galhardoni R, de Andrade DC (2019). Prevalence of chronic pain in developing countries: systematic review and meta-analysis. Pain Rep.

[10] Elzahaf RA, Tashani OA, Unsworth BA, Johnson MI (2012). The prevalence of chronic pain with an analysis of countries with a Human Development Index less than 0.9: a systematic review without meta-analysis. Curr Med Res Opin.

[11] Carr DB (2016). "Pain is a public health problem" --what does that mean and why should we care?. Pain Med.

[12] Goldberg DS, McGee SJ (2011). Pain as a global public health priority. BMC Public Health.

[13] Sarzi-Puttini P, Vellucci R, Zuccaro SM, Cherubino P, Labianca R, Fornasari D (2012). The appropriate treatment of chronic pain. Clin Drug Investig.

[14] Edwards RR, Dworkin RH, Sullivan MD, Turk DC, Wasan AD (2016). The role of psychosocial processes in the development and maintenance of chronic pain. J Pain.

[15] Engel GL (1977). The need for a new medical model: a challenge for biomedicine. Science.

[16] Devan H, Hale L, Hempel D, Saipe B, Perry MA (2018). What works and does not work in a self-management intervention for people with chronic pain? Qualitative systematic review and meta-synthesis. Phys Ther.

[17] Shery M, MacNeil C (2004). Peer support: what makes it unique?. Int J Psychosocial Rehabil.

[18] Nicholas MK, Asghari A, Blyth FM, Wood BM, Murray R, McCabe R, Brnabic A, Beeston L, Corbett M, Sherrington C, Overton S (2017). Long-term outcomes from training in self-management of chronic pain in an elderly population: a randomized controlled trial. Pain.

[19] Bartone PT, Bartone JV, Violanti JM, Gileno ZM (2019). Peer support services for bereaved survivors: a systematic review. Omega (Westport).

[20] Dennis CL (2003). Peer support within a health care context: a concept analysis. Int J Nurs Stud.

[21] Farr M, Brant H, Patel R, Linton MJ, Ambler N, Vyas S, Wedge H, Watkins S, Horwood J (2021). Experiences of patient-led chronic pain peer support groups after pain management programs: a qualitative study. Pain Med.

[22] Pester BD, Tankha H, Caño A, Tong S, Grekin E, Bruinsma J, Gootee J, Lumley MA (2022). Facing pain together: a randomized controlled trial of the effects of Facebook support groups on adults with chronic pain. J Pain.

[23] Stenberg N, Gillison F, Rodham K (2022). How do peer support interventions for the self-management of chronic pain, support basic psychological needs? A systematic review and framework synthesis using self-determination theory. Patient Educ Couns.

[24] Tolley JA, Michel MA, Williams AE, Renschler JS (2020). Peer support in the treatment of chronic pain in adolescents: a review of the literature and available resources. Children (Basel).

[25] Naslund JA, Grande SW, Aschbrenner KA, Elwyn G (2014). Naturally occurring peer support through social media: the experiences of individuals with severe mental illness using YouTube. PLoS One.

[26] Daynes-Kearney R, Gallagher S (2023). Online support groups for family caregivers: scoping review. J Med Internet Res.

[27] Hossain SN, Jaglal SB, Shepherd J, Perrier L, Tomasone JR, Sweet SN, Luong D, Allin S, Nelson ML, Guilcher SJ, Munce SE (2021). Web-based peer support interventions for adults living with chronic conditions: scoping review. JMIR Rehabil Assist Technol.

[28] Merolli M, Gray K, Martin-Sanchez F (2016). Patient participation in chronic pain management through social media: a clinical study. Stud Health Technol Inform.

[29] Brennen JS, Simon F, Howard PN, Nielsen RK (2020). Types, sources, and claims of COVID-19 misinformation. Reuters Institute for the Study of Journalism.

[30] Suarez-Lledo V, Alvarez-Galvez J (2021). Prevalence of health misinformation on social media: systematic review. J Med Internet Res.

[31] Ziemer KS, Fuhrmann A, Hoffman MA (2017). Effectiveness of a positive writing intervention for chronic pain: a randomized trial. Myopain.

[32] Guillory J, Chang P, Henderson CR Jr, Shengelia R, Lama S, Warmington M, Jowza M, Waldman S, Gay G, Reid MC (2015). Piloting a text message-based social support intervention for patients with chronic pain: establishing feasibility and preliminary efficacy. Clin J Pain.

[33] Caes L, Jones A, Jordan A (2018). Engaging use of social media as a research tool to capture the daily life experiences of young people with chronic pain. Evid Based Nurs.

[34] Cooper K, Kirkpatrick P, Wilcock S (2014). The effectiveness of peer support interventions for community-dwelling adults with chronic non-cancer pain: a systematic review. JBI Database System Rev Implement Rep.

[35] Palant A, Himmel W (2019). Are there also negative effects of social support? A qualitative study of patients with inflammatory bowel disease. BMJ Open.

[36] Gómez Penedo JM, Rubel JA, Blättler L, Schmidt SJ, Stewart J, Egloff N, Grosse Holtforth M (2020). The complex interplay of pain, depression, and anxiety symptoms in patients with chronic pain: a network approach. Clin J Pain.

[37] Lee H, Hübscher M, Moseley GL, Kamper SJ, Traeger AC, Mansell G, McAuley JH (2015). How does pain lead to disability? A systematic review and meta-analysis of mediation studies in people with back and neck pain. Pain.

[38] Lerman SF, Rudich Z, Brill S, Shalev H, Shahar G (2015). Longitudinal associations between depression, anxiety, pain, and pain-related disability in chronic pain patients. Psychosom Med.

[39] Knuppenburg RM, Fredericks CM (2021). Linguistic affect: positive and negative emotion words are contagious, predict likability, and moderate positive and negative affect. Inq J.

[40] Blyth FM, Macfarlane GJ, Nicholas MK (2007). The contribution of psychosocial factors to the development of chronic pain: the key to better outcomes for patients?. Pain.

[41] Gatchel RJ, McGeary DD, McGeary CA, Lippe B (2014). Interdisciplinary chronic pain management: past, present, and future. Am Psychol.

[42] Diatchenko L, Fillingim RB, Smith SB, Maixner W (2013). The phenotypic and genetic signatures of common musculoskeletal pain conditions. Nat Rev Rheumatol.

[43] Şentürk İA, Şentürk E, Üstün I, Gökçedağ A, Yıldırım NP, İçen NK (2023). High-impact chronic pain: evaluation of risk factors and predictors. Korean J Pain.

[44] Wager J, Brown D, Kupitz A, Rosenthal N, Zernikow B (2020). Prevalence and associated psychosocial and health factors of chronic pain in adolescents: differences by sex and age. Eur J Pain.

[45] Hall AM, Kamper SJ, Maher CG, Latimer J, Ferreira ML, Nicholas MK (2011). Symptoms of depression and stress mediate the effect of pain on disability. Pain.

[46] Ganster DC, Victor B (1988). The impact of social support on mental and physical health. Br J Med Psychol.

[47] Hale CJ, Hannum JW, Espelage DL (2010). Social support and physical health: the importance of belonging. J Am Coll Health.

[48] Kelly ME, Duff H, Kelly S, McHugh Power JE, Brennan S, Lawlor BA, Loughrey DG (2017). The impact of social activities, social networks, social support and social relationships on the cognitive functioning of healthy older adults: a systematic review. Syst Rev.

[49] Vandervoort D (1999). Quality of social support in mental and physical health. Curr Psychol.

[50] Holahan CJ, Moos RH (1981). Social support and psychological distress: a longitudinal analysis. J Abnorm Psychol.

[51] Lepore SJ (1992). Social conflict, social support, and psychological distress: evidence of cross-domain buffering effects. J Pers Soc Psychol.

[52] Menec VH, Newall NE, Mackenzie CS, Shooshtari S, Nowicki S (2020). Examining social isolation and loneliness in combination in relation to social support and psychological distress using Canadian Longitudinal Study of Aging (CLSA) data. PLoS One.

[53] Uysal A, Ascigil E, Turunc G (2017). Spousal autonomy support, need satisfaction, and well-being in individuals with chronic pain: a longitudinal study. J Behav Med.

[54] Abbasi M, Dehghani M, Keefe FJ, Jafari H, Behtash H, Shams J (2012). Spouse-assisted training in pain coping skills and the outcome of multidisciplinary pain management for chronic low back pain treatment: a 1-year randomized controlled trial. Eur J Pain.

[55] Keefe FJ, Caldwell DS, Baucom D, Salley A, Robinson E, Timmons K, Beaupre P, Weisberg J, Helms M (1996). Spouse-assisted coping skills training in the management of osteoarthritic knee pain. Arthritis Care Res.

[56] Craig KD (2009). The social communication model of pain. Can Psychol.

[57] Hadjistavropoulos T, Craig KD, Duck S, Cano A, Goubert L, Jackson PL, Mogil JS, Rainville P, Sullivan MJ, Williams AC, Vervoort T, Fitzgerald TD (2011). A biopsychosocial formulation of pain communication. Psychol Bull.

[58] Sullivan MJ (2012). The communal coping model of pain catastrophising: clinical and research implications. Can Psychol.

[59] Craig KD (2015). Social communication model of pain. Pain.

[60] Lapidot-Lefler N, Barak A (2012). Effects of anonymity, invisibility, and lack of eye-contact on toxic online disinhibition. Comput Hum Behav.

[61] Malik S, Coulson NS (2011). The therapeutic potential of the internet: exploring self-help processes in an internet forum for young people with inflammatory bowel disease. Gastroenterol Nurs.

[62] Omernick E, Sood SO (2013). The impact of anonymity in online communities. Proceedings of the International Conference on Social Computing.

[63] Carr CT, Hayes RA (2015). Social media: defining, developing, and divining. Atl J Commun.

[64] Kaplan AM, Haenlein M (2010). Users of the world, unite! The challenges and opportunities of social media. Bus Horiz.

[65] Landro L (1999). Alone together. Cancer patients and survivors find treatment--and support--online. It can make all the difference. Oncologist.

[66] Eysenbach G, Sa ER, Diepgen TL (1999). Shopping around the internet today and tomorrow: towards the millennium of cyber medicine. BMJ.

[67] Frank SR (2000). Digital health care--the convergence of health care and the internet. J Ambul Care Manage.

[68] Eysenbach G, Powell J, Englesakis M, Rizo C, Stern A (2004). Health related virtual communities and electronic support groups: systematic review of the effects of online peer to peer interactions. BMJ.

[69] Bennett GG, Glasgow RE (2009). The delivery of public health interventions via the internet: actualizing their potential. Annu Rev Public Health.

[70] Huh J, Kwon BC, Kim SH, Lee S, Choo J, Kim J, Choi MJ, Yi JS (2016). Personas in online health communities. J Biomed Inform.

[71] Ashtari S, Taylor J, Lai G (2020). Systematic review of social networking support groups for genetic-disorders pain management. Proceedings of the 53rd Hawaii International Conference on System Sciences.

[72] Dhar VK, Kim Y, Graff JT, Jung AD, Garrett J, Dick LE, Harris J, Shah SA (2018). Benefit of social media on patient engagement and satisfaction: results of a 9-month, qualitative pilot study using Facebook. Surgery.

[73] Tsai S, Crawford E, Strong J (2018). Seeking virtual social support through blogging: a content analysis of published blog posts written by people with chronic pain. Digit Health.

[74] Davidson L, Chinman M, Kloos B, Weingarten R, Stayner D, Tebes JK (1999). Peer support among individuals with severe mental illness: a review of the evidence. Clin Psychol Sci Pract.

[75] Hall SL, Ryan DJ, Beatty J, Grubbs L (2015). Recommendations for peer-to-peer support for NICU parents. J Perinatol.

[76] Kingod N, Cleal B, Wahlberg A, Husted GR (2017). Online peer-to-peer communities in the daily lives of people with chronic illness: a qualitative systematic review. Qual Health Res.

[77] Merolli M, Gray K, Martin-Sanchez F (2013). Health outcomes and related effects of using social media in chronic disease management: a literature review and analysis of affordances. J Biomed Inform.

[78] Ruehlman L, Karoly P, Enders C (2012). A randomized controlled evaluation of an online chronic pain self management program. Pain.

[79] Trudeau KJ, Pujol LA, DasMahapatra P, Wall R, Black RA, Zacharoff K (2015). A randomized controlled trial of an online self-management program for adults with arthritis pain. J Behav Med.

[80] Meldrum S, Savarimuthu BT, Licorish S, Tahir A, Bosu M, Jayakaran P (2017). Is knee pain information on YouTube videos perceived to be helpful? An analysis of user comments and implications for dissemination on social media. Digit Health.

[81] Rod K (2016). Finding ways to lift barriers to care for chronic pain patients: outcomes of using internet-based self-management activities to reduce pain and improve quality of life. Pain Res Manag.

[82] Willis E (2016). Patients' self-efficacy within online health communities: facilitating chronic disease self-management behaviors through peer education. Health Commun.

[83] Berkanish P, Pan S, Viola A, Rademaker Q, Devine KA (2022). Technology-based peer support interventions for adolescents with chronic illness: a systematic review. J Clin Psychol Med Settings.

[84] Zhang R (2017). The stress-buffering effect of self-disclosure on Facebook: an examination of stressful life events, social support, and mental health among college students. Comput Hum Behav.

[85] Young SD, Koussa M, Lee SJ, Perez H, Gill N, Gelberg L, Heinzerling K (2018). Feasibility of a social media/online community support group intervention among chronic pain patients on opioid therapy. J Addict Dis.

[86] What is Reddit?. Reddit.

[87] Goudman L, de Smedt A, Moens M (2022). Social media and chronic pain: what do patients discuss?. J Pers Med.

[88] Hardeniya T, Borikar DA (2016). Dictionary based approach to sentiment analysis - a review. Int J Adv Eng Manag Sci.

[89] Birjali M, Kasri M, Beni-Hssane A (2021). A comprehensive survey on sentiment analysis: approaches, challenges and trends. Knowl Based Syst.

[90] Wankhade M, Rao AC, Kulkarni C (2022). A survey on sentiment analysis methods, applications, and challenges. Artif Intell Rev.

[91] Grinsted A, Moore JC, Jevrejeva S (2004). Application of the cross wavelet transform and wavelet coherence to geophysical time series. Nonlin Processes Geophys.

[92] Issartel J, Bardainne T, Gaillot P, Marin L (2015). The relevance of the cross-wavelet transform in the analysis of human interaction - a tutorial. Front Psychol.

[93] Baumgartner J, Zannettou S, Keegan B, Squire M, Blackburn J (2020). The Pushshift Reddit dataset. Proc Int AAAI Conf Web Soc Media.

[94] Amaya A, Bach R, Keusch F, Kreuter F (2019). New data sources in social science research: things to know before working with Reddit data. Soc Sci Comput Rev.

[95] Necaise A, Han J, Vrzáková H, Amon MJ (2023). Understanding collective human behavior in social media networks via the dynamical hypothesis: applications to radicalization and conspiratorial beliefs. Top Cogn Sci.

[96] Davcheva E, Adam M, Benlian A (2019). User dynamics in mental health forums – a sentiment analysis perspective. Proceedings of 14th International Conference on Wirtschaftsinformatik.

[97] Zhang S, Bantum E, Owen J, Elhadad N (2014). Does sustained participation in an online health community affect sentiment?. AMIA Annu Symp Proc.

[98] Pennebaker JW, Francis ME, Booth RJ (2001). Linguistic inquiry and word count: LIWC 2001.

[99] de Choudhury M (2015). Anorexia on Tumblr: a characterization study. Proceedings of the 5th International Conference on Digital Health 2015.

[100] del Pilar Salas-Zárate M, López-López E, Valencia-García R, Aussenac-Gilles N, Almela Á, Alor-Hernández G (2014). A study on LIWC categories for opinion mining in Spanish reviews. J Inf Sci.

[101] Sharma C, Whittle S, Haghighi PD, Burstein F, Keen H (2020). Sentiment analysis of social media posts on pharmacotherapy: a scoping review. Pharmacol Res Perspect.

[102] Wang Y, Weber I, Mitra P (2016). Quantified self meets social media: sharing of weight updates on Twitter. Proceedings of the 6th International Conference on Digital Health Conference.

[103] Monzani D, Vergani L, Pizzoli SF, Marton G, Pravettoni G (2021). Emotional tone, analytical thinking, and somatosensory processes of a sample of Italian tweets during the first phases of the COVID-19 pandemic: observational study. J Med Internet Res.

[104] Hutto CJ, Gilbert E (2014). VADER: a parsimonious rule-based model for sentiment analysis of social media text. Proceedings of the Eighth International AAAI Conference on Weblogs and Social Media.

[105] Elbagir S, Yang J (2019). Twitter sentiment analysis using natural language toolkit and VADER sentiment. Proceedings of the International MultiConference of Engineers and Computer Scientists 2019.

[106] Shiffman S, Stone AA, Hufford MR (2008). Ecological momentary assessment. Annu Rev Clin Psychol.

[107] Torrence C, Compo GP (1998). A practical guide to wavelet analysis. Bull Am Meteorol Soc.

[108] Gouhier TC, Grinsted A, Simko V (2019). R package biwavelet: conduct univariate and bivariate wavelet analyses. GitHub.

[109] Zar JH (1999). Biostatistical Analysis.

[110] Lakens D (2013). Calculating and reporting effect sizes to facilitate cumulative science: a practical primer for t-tests and ANOVAs. Front Psychol.

[111] Reddan MC, Young H, Falkner J, López-Solà M, Wager TD (2020). Touch and social support influence interpersonal synchrony and pain. Soc Cogn Affect Neurosci.

[112] Necaise A, Williams A, Vrzakova H, Amon MJ (2021). Regularity versus novelty of users' multimodal comment patterns and dynamics as markers of social media radicalization. Proceedings of the 32nd ACM Conference on Hypertext and Social Media.

[113] Wood A, Lipson J, Zhao O, Niedenthal P, Robinson MD, Thomas LE (2021). Forms and functions of affective synchrony. Handbook of Embodied Psychology.

[114] Doré BP, Morris RR (2018). Linguistic synchrony predicts the immediate and lasting impact of text-based emotional support. Psychol Sci.

[115] Ireland ME, Slatcher RB, Eastwick PW, Scissors LE, Finkel EJ, Pennebaker JW (2011). Language style matching predicts relationship initiation and stability. Psychol Sci.

[116] Ramseyer FT, Tschacher W, Rossler OE, Vrobel S, Marks-Tarlow T (2008). Synchrony in dyadic psychotherapy sessions. Simultaneity: Temporal Structures And Observer Perspectives.

[117] Mogan R, Fischer R, Bulbulia JA (2017). To be in synchrony or not? A meta-analysis of synchrony's effects on behavior, perception, cognition and affect. J Exp Soc Psychol.

[118] Tschacher W, Rees GM, Ramseyer F (2014). Nonverbal synchrony and affect in dyadic interactions. Front Psychol.

[119] Matthias MS, Kukla M, McGuire AB, Bair MJ (2016). How do patients with chronic pain benefit from a peer-supported pain self-management intervention? A qualitative investigation. Pain Med.

[120] Bandura A (1977). Social Learning Theory.

[121] Areli E, Godfrey HK, Perry MA, Hempel D, Saipe B, Grainger R, Hale L, Devan H (2021). 'I think there is nothing . . . that is really comprehensive': healthcare professionals' views on recommending online resources for pain self-management. Br J Pain.

[122] Devan H, Godfrey HK, Perry MA, Hempel D, Saipe B, Hale L, Grainger R (2019). Current practices of health care providers in recommending online resources for chronic pain self-management. J Pain Res.

[123] Sannon S, Murnane E, Bazarova N, Gay G (2019). "I was really, really nervous posting it": communicating about invisible chronic illnesses across social media platforms. Proceedings of the 2019 CHI Conference on Human Factors in Computing Systems.

[124] Pascual-Ferrá P, Alperstein N, Barnett DJ, Rimal RN (2021). Toxicity and verbal aggression on social media: polarized discourse on wearing face masks during the COVID-19 pandemic. Big Data Soc.

[125] Zaheri S, Leath J, Stroud D (2020). Toxic comment classification. SMU Data Sci Rev.

[126] Stevens HR, Acic I, Rhea S (2021). Natural language processing insight into LGBTQ+ youth mental health during the COVID-19 pandemic: longitudinal content analysis of anxiety-provoking topics and trends in emotion in LGBTeens Microcommunity Subreddit. JMIR Public Health Surveill.

[127] Gatchel RJ, Neblett R, Kishino N, Ray CT (2016). Fear-avoidance beliefs and chronic pain. J Orthop Sports Phys Ther.

[128] Carstens JK, Shaw WS, Boersma K, Reme SE, Pransky G, Linton SJ (2014). When the wind goes out of the sail - declining recovery expectations in the first weeks of back pain. Eur J Pain.

[129] Ferro MA, Boyle MH (2013). Longitudinal invariance of measurement and structure of global self-concept: a population-based study examining trajectories among adolescents with and without chronic illness. J Pediatr Psychol.

[130] Glette M, Stiles TC, Borchgrevink PC, Landmark T (2020). The natural course of chronic pain in a general population: stability and change in an eight-wave longitudinal study over four years (the HUNT pain study). J Pain.

[131] Karels CH, Bierma-Zeinstra SM, Burdorf A, Verhagen AP, Nauta AP, Koes BW (2007). Social and psychological factors influenced the course of arm, neck and shoulder complaints. J Clin Epidemiol.

[132] Vlaeyen JW, Linton SJ (2000). Fear-avoidance and its consequences in chronic musculoskeletal pain: a state of the art. Pain.

[133] Crombez G, Eccleston C, van Damme S, Vlaeyen JW, Karoly P (2012). Fear-avoidance model of chronic pain: the next generation. Clin J Pain.

[134] Zale EL, Ditre JW (2015). Pain-related fear, disability, and the fear-avoidance model of chronic pain. Curr Opin Psychol.

[135] Sippel LM, Pietrzak RH, Charney DS, Mayes LC, Southwick SM (2015). How does social support enhance resilience in the trauma-exposed individual?. Ecol Soc.

[136] Wilks SE (2008). Resilience amid academic stress: the moderating impact of social support among social work students. Adv Soc Work.

[137] Gonzalez-Polledo E, Tarr J (2016). The thing about pain: the remaking of illness narratives in chronic pain expressions on social media. New Media Soc.

[138] Sendra A, Farré J (2020). Communicating the experience of chronic pain through social media: patients’ narrative practices on Instagram. J Commun Healthc.

[139] Bevers K, Watts L, Kishino ND, Gatchel RJ (2016). The biopsychosocial model of the assessment, prevention, and treatment of chronic pain. US Neurol.

[140] Vlaeyen JW, Haslbeck JM, Sjouwerman R, Peters ML (2022). Towards a dynamic account of chronic pain. Pain.

[141] Broderick JE, Junghaenel DU, Schwartz JE (2005). Written emotional expression produces health benefits in fibromyalgia patients. Psychosom Med.

[142] Gillis ME, Lumley MA, Mosley-Williams A, Leisen JC, Roehrs T (2006). The health effects of at-home written emotional disclosure in fibromyalgia: a randomized trial. Ann Behav Med.

[143] Junghaenel DU, Schwartz JE, Broderick JE (2010). Differential efficacy of written emotional disclosure for subgroups of fibromyalgia patients. Brit J Health Psychol.

[144] Nils F, Rimé B (2012). Beyond the myth of venting: social sharing modes determine the benefits of emotional disclosure. Eur J Soc Psychol.

[145] Zhan J, Xu H, Ren J, Luo J (2020). Is catharsis beneficial or harmful? The psychological intervention effect and potential harm of catharsis. Adv Psychol Sci.

[146] Prieto-Callejero B, Gómez-Salgado J, Alvarado-Gómez F, Dias A, García-Iglesias JJ, Ruiz-Frutos C (2020). [Systematic review of the reduction of negative emotional effects in emergency and disaster response workers through catharsis techniques]. Arch Prev Riesgos Labor.

